# From prohibition to integration: LLMs in scientific publishing

**DOI:** 10.3389/fgene.2026.1947911

**Published:** 2026-09-22

**Authors:** Boris Kirov, Aleksandar Marinchev, Slavil Peykov, Vassil Galabov

**Affiliations:** 1 BioInfoTech Lab, Research and Development and Innovation Consortium, Sofia, Bulgaria; 2 Faculty of German Engineering Education and Industrial Management (FDIBA), TU – Sofia, Sofia, Bulgaria; 3 Faculty of Automatics, TU – Sofia, Sofia, Bulgaria; 4 Faculty of Biology, Sofia University St. Kliment Ohridski, Sofia, Bulgaria

**Keywords:** artificial intelligence, epistemic integrity, peer review, research integrity, scientific authorship, scientific publishing

## Abstract

The growing use of large language models (LLMs) in scientific writing has intensified debates about authorship, originality, and research integrity. Critics often argue that LLM-generated text lacks originality because it is derived from existing literature. This Perspective challenges that assumption by arguing that scientific knowledge, whether produced by humans or machines, is inherently cumulative. We argue that both researchers and LLM systems operate within inherited bodies of scientific knowledge, making intellectual leadership rather than textual production the defining criterion of authorship. Researchers remain the legitimate authors when they formulate the research question, develop the central ideas, and critically direct LLMs throughout the writing process. Beyond authorship, we argue that scientific publishing increasingly rewards procedural compliance over epistemic quality, allowing formal citation practices to substitute for conceptual coherence. Rather than undermining research integrity, LLMs offer an opportunity to restore greater emphasis on reasoning and critical evaluation. Finally, we propose integrating standardized LLM-based verification directly into journal submission systems. Automated verification of references, factual consistency, and scientific and technical integrity should precede peer review, allowing reviewers to focus on originality, scientific significance, and conceptual evaluation. Properly governed, LLMs can become not only a writing assistant but also an institutional component of scientific quality assurance.

## 1 Introduction: a new front in the authorship debate

The emergence of large language models (LLMs) has transformed scientific writing and reignited debates over authorship, originality, and research integrity. Critics argue that LLM-generated text is inherently unoriginal because it is derived from existing literature. Yet scientific knowledge has always been cumulative. Scientific knowledge advances cumulatively, with new contributions extending existing concepts, methods, and observations (Jo et al., 2022).

The debate also unfolds amid a broader crisis of confidence in scientific publishing. Only 15.1% of results in a study of 93 fMRI-based statistics papers could be reproduced using the available code and data (Xiong and Cribben, 2023, p. 115), while more than half of respondents in Nature’s international survey reported difficulties reproducing even their own work (Baker, 2016, p. 452). In addition, more than 10,000 research papers were retracted in 2023 alone, a record high, with paper mills and compromised publishing practices contributing substantially to this trend (Van Noorden, 2023, pp. 479–481; Else and Van Noorden, 2021, pp. 516–519; Byrne and Christopher, 2020, p. 583). Together, these observations reflect broader concerns about reproducibility and research quality that have been discussed extensively in the scientific literature (Begley and Ioannidis, 2015, pp. 116–118). At the same time, research assessment has become increasingly dependent on publication counts, citation metrics, and journal indicators that were never intended to serve as direct measures of scientific quality (Hicks et al., 2015, pp. 429–431).

Within this environment, LLMs appear simultaneously as a threat and an opportunity. Large language models may fabricate references or unsupported claims, with studies reporting that 69% of references generated in response to medical questions were fabricated (Gravel et al., 2023, pp. 226). These concerns are legitimate, but they also raise a broader question. If science has always progressed by building on previous knowledge, are LLMs fundamentally different from the cumulative processes through which human researchers develop ideas?

This Perspective argues that it is not. Scientific authorship is defined by intellectual leadership rather than by the physical act of writing. Authorship begins long before the first sentence is written. Researchers define the scientific problem, formulate hypotheses, design the study, determine which data should be collected, select appropriate methods, interpret the resulting evidence, and accept responsibility for the conclusions. LLMs may assist in communicating those decisions, but they neither make them nor assume responsibility for them. The deeper challenge lies elsewhere: contemporary publishing increasingly rewards formal compliance over conceptual quality. The latter often consumes substantial time and effort that could otherwise be devoted to scientific reasoning, experimental design, and the interpretation of results. At the same time, publishers already rely on automated tools to support editorial quality control, including plagiarism detection, image integrity screening, reference validation, and other forms of technical assessment. These technologies demonstrate that automated quality assurance is already an accepted component of scientific publishing. Extending this existing infrastructure to include LLM-assisted verification therefore represents an incremental evolution of current editorial practice rather than a fundamentally new departure. Building on this argument, we propose that LLMs should become part of the scientific quality assurance process itself. Standardized LLM-based verification integrated into journal submission systems could provide a consistent first layer of editorial assessment, automatically verifying the scientific and technical integrity and consistency of both author- and LLM-generated content before peer review, thereby enabling peer reviewers to concentrate on originality, scientific significance, and conceptual evaluation rather than routine technical verification.

## 2 Intellectual inheritance in science

Science has never advanced through isolated acts of creation. Every scientific contribution builds upon previously established concepts, methods, and observations, reflecting the cumulative nature of scientific knowledge (Jo et al., 2022). Scientific originality therefore lies not in creating knowledge *ex nihilo*, but in extending existing knowledge through new questions, interpretations, methods, or empirical evidence.

Scientific writing follows the same pattern. Before making an original contribution, researchers spend years learning the language, methods, and conventions of their disciplines. Large language models acquire knowledge differently, yet they also generate text from accumulated scientific literature rather than from independent invention.

Seen from this perspective, the common criticism that LLM-assisted writing is inherently derivative loses much of its force. Human scholarship is derivative in the same fundamental sense: every manuscript depends on previous knowledge. What distinguishes scientific authorship is not the novelty of the wording but the intellectual work behind scientific discovery: defining worthwhile questions, designing appropriate methods, generating new empirical evidence, interpreting results, and constructing coherent explanations. The manuscript records this process; it does not constitute it. LLMs may assist in writing, but the scientific investigation itself remains a human responsibility.

Human researchers and LLMs therefore occupy similar epistemic positions. Both rely on accumulated scientific knowledge. The difference lies not in the source of that knowledge but in agency. Researchers define the scientific problem, evaluate competing interpretations, and accept responsibility for the conclusions.

This perspective also helps explain a common criticism of LLMs: because they generate responses from statistical regularities in existing knowledge, their outputs may be less diverse than those produced by human researchers and may therefore be less likely to produce genuinely unconventional conceptual breakthroughs. Recent empirical work has indeed found that LLM-generated creative outputs are substantially more homogeneous than human-generated ones (Wenger and Kenett, 2026), while other studies show important differences between human and LLM creativity rather than simple equivalence (Wang et al., 2026).

The relevant question is not whether LLMs transcend existing knowledge, but whether they enable researchers to formulate better questions, interpret evidence more effectively, and communicate scientific ideas more clearly. If intellectual direction remains firmly in human hands, LLMs change neither the nature of scientific authorship nor the source of scientific originality. They change only the means through which scientific knowledge is developed and communicated.

## 3 Rebutting the common objections

The debate over LLM-assisted scientific writing has largely focused on three recurring concerns: authorship and accountability, the originality of LLM-generated content, and the potential erosion of trust and transparency in scientific communication (Thorp, 2023, p. 313; Nature Editorial, 2023, p. 612; COPE, 2023, “Authorship and AI tools”). None of these concerns, however, is unique to LLMs.

The plagiarism argument assumes that originality requires entirely novel expression. Scientific writing has never met that standard. Every manuscript builds upon previous research, employs established scientific terminology, and positions its contribution within an existing body of knowledge (de Solla Price, 1963). What distinguishes scientific work is not novel wording but original questions, interpretations, and conclusions. When researchers define the problem, evaluate the evidence, and critically shape the argument, they remain the authors regardless of the tools used to produce the text.

The same applies to creativity. LLMs recombine existing knowledge, but so do scientists. New ideas rarely emerge from independent invention; more often they arise by connecting familiar concepts in unexpected ways (Simonton, 2021, pp. 1–2). LLMs may accelerate this process, but only researchers can judge whether those connections are scientifically meaningful.

Perhaps the strongest concern is trust in the integrity of the scientific record. Large language models may generate fabricated references or unsupported claims (Gravel et al., 2023, p. 226). Yet fraudulent publications, paper mills, manipulated images, and fabricated data all long predate LLMs (Rossner and Yamada, 2004, pp. 11–15; Cromey, 2010, pp. 639–667; Else and Van Noorden, 2021, pp. 516–519; Van Noorden, 2023, pp. 479–481). The problem is therefore not LLMs themselves but inadequate verification.

The appropriate response is not to reject LLMs but to apply the same standards of accountability that govern every scientific instrument. Human authors remain responsible for verifying all claims before publication. Under these conditions, LLMs introduce no fundamentally new ethical problem; they simply become another research tool whose value depends on the integrity of those who use it.

## 4 Citation fetishism and epistemic integrity

Citation is indispensable to scientific communication. It acknowledges intellectual debt, enables verification, and places new work within existing knowledge. Increasingly, however, citation has come to function not only as documentation but also as a proxy for scientific legitimacy.

This shift has important consequences. Scientific claims in many contexts are evaluated first by whether they are accompanied by published references rather than by whether they are logically coherent or empirically plausible. Formal attribution can gradually replace critical evaluation.

The growing number of retractions, fraudulent publications, and commercial papermills illustrates the limitations of this model (Else and Van Noorden, 2021, pp. 516–518; Byrne and Christopher, 2020, pp. 15–18). Correct citation is essential, but it cannot establish scientific validity. References indicate where ideas originate; they do not determine whether those ideas are correct.

Paradoxically, LLMs offer an opportunity to restore this distinction. By reducing the effort required to produce technically compliant manuscripts, LLMs allow researchers to devote more attention to scientific reasoning, interpretation, and conceptual development. Properly used, LLMs challenge not citation itself but the disproportionate importance citation has acquired within contemporary academic culture.

## 5 LLMs as a corrective force

The rapid adoption of LLMs reflects more than technological progress. It exposes a structural imbalance in scientific publishing. Researchers spend increasing amounts of time meeting editorial requirements that contribute little to scientific discovery.

By automating routine tasks, LLMs allow researchers to redirect attention toward the intellectual core of research. Scientific value should depend on the originality of the questions asked, the quality of the evidence presented, and the coherence of the arguments developed—not on the effort invested in producing technically flawless manuscripts.

LLMs may also broaden participation in science. Researchers working in under-resourced environments or writing in a second language can more readily meet the formal expectations of international journals, reducing barriers that have long limited participation in scholarly communication.

This does not diminish human responsibility. LLMs change how science is communicated, not who is accountable for it. Used responsibly, they offer an opportunity to restore emphasis on scientific reasoning rather than procedural compliance.

## 6 Normative guidelines for LLM-assisted authorship

If LLMs are to become a permanent part of scientific publishing, their use should be governed by institutional principles rather than *ad hoc* editorial policies. The challenge is not technological but sociological: scientific institutions must adapt without weakening the norms that sustain trust in scientific knowledge. Merton identified universalism and organized skepticism as core principles through which scientific claims are evaluated according to impersonal rather than personal criteria (Merton, 1973, pp. 270–273). LLMs should reinforce these principles, not replace them.

LLM-assisted authorship should follow four principles: transparency, responsibility, verification, and purpose. The use of LLMs should be disclosed when it contributes substantially to manuscript preparation. Human authors remain fully accountable for every statement and conclusion. References, factual claims, figures, and numerical results must be verified before publication. Finally, LLMs should improve scientific communication rather than compensate for weak reasoning or inflate publication output.

These principles, however, should not depend entirely on individual diligence. Scientific publishing already relies on automated plagiarism detection, bibliographic validation, and image integrity screening (Crossref, 2026, “Similarity Check”; Elsevier, 2026, “Plagiarism detection”; Rossner and Yamada, 2004, pp. 11–15; Cromey, 2010, pp. 641–647). LLM-assisted authorship should follow the same institutional logic.

We therefore propose integrating standardized LLM-based verification directly into journal submission systems. Before peer review, manuscripts should undergo automated verification of references, factual claims, internal consistency, figures, and scientific and technical integrity. Such systems would not judge originality or scientific merit. Their role would be to provide a consistent first layer of quality assurance applied equally to every submission.

This approach also addresses an important weakness of the current publication process. Detecting fabricated references, unsupported claims, or technical inconsistencies currently depends largely on the expertise, available time, and subjective judgement of individual reviewers. Automated verification cannot replace peer review, but it can ensure that every manuscript receives the same initial scrutiny before reaching human experts. Reviewers can then concentrate on what only they can evaluate: originality, scientific significance, conceptual coherence, and interpretation of evidence.

Properly implemented, LLMs become more than a writing assistant. They become part of the institutional infrastructure that supports transparency, consistency, and trust in scientific publishing. Finally, LLM-based verification should itself be transparent, version-controlled, and subject to continuous human oversight.

The proposed workflow is summarized in Figure 1.

**FIGURE 1:**
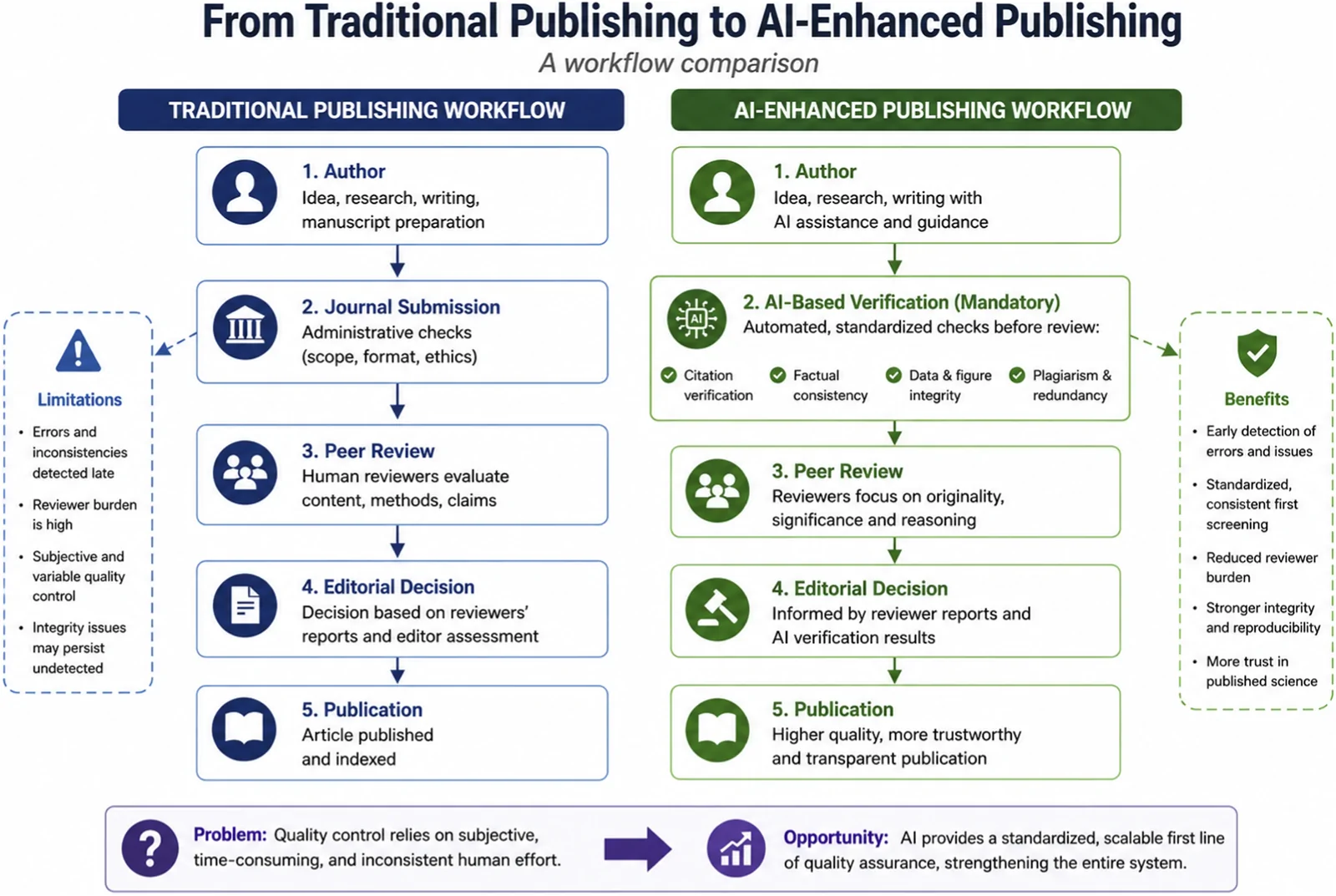
Proposed integration of standardized LLM-based verification into the scientific publishing workflow.

## 7 Discussion

The debate over LLM-assisted authorship is often framed as a question of whether machines can write scientific papers. We believe this is the wrong question. Scientific progress has never depended primarily on producing text but on asking important questions, evaluating evidence critically, and developing convincing explanations. The manuscript is the product of scientific inquiry, not the inquiry itself.

From this perspective, LLMs do not redefine scientific authorship. Scientific work begins with identifying a problem and continues through study design, data collection, analysis, interpretation, and critical judgement. Writing is only the final stage of that process. LLMs may assist in communicating scientific knowledge, but responsibility for every preceding stage remains entirely with the researcher. Authorship is therefore determined by intellectual leadership rather than by the physical act of writing.

The same reasoning applies to scientific publishing. If LLMs assist researchers in preparing manuscripts, they should also assist journals in evaluating their scientific and technical integrity. Standardized LLM-based verification should become the first stage of editorial assessment, allowing peer reviewers to focus on originality, scientific significance, and conceptual quality rather than routine technical checks.

The widespread adoption of LLMs provides an opportunity to reassess the balance between procedural requirements and scientific reasoning. Over recent decades, increasing effort has been devoted to procedural and editorial requirements that, while necessary, do not themselves generate knowledge. LLMs offer an opportunity to reverse that trend by shifting routine verification from researchers and reviewers to standardized computational systems.

Science has never advanced because someone wrote better sentences. Science advances because better questions produce better evidence and better explanations. If LLMs help scientists devote more attention to that process, they will have strengthened not only scientific writing but science itself.

The central ethical concern is therefore not the use of LLMs to assist with writing, but the replacement of human intellectual leadership by automated reasoning. Scientific manuscripts derive their value from the originality of the questions they address, the appropriateness of their methodology, the validity of their interpretation, and the significance of their conclusions—not from the process by which individual sentences are composed. If authors delegate these core intellectual responsibilities to an LLM, the resulting work ceases to represent genuine scientific authorship regardless of the linguistic quality of the manuscript. By contrast, when LLMs are used solely to improve communication while scientific reasoning remains under human direction, no fundamental challenge to authorship arises. Determining whether a manuscript contains genuine intellectual contribution rather than merely technically competent prose remains an inherently human task and lies at the heart of peer review. Automated verification can assess scientific and technical integrity, but only expert reviewers can evaluate scientific originality, conceptual depth, and the relevance of the work to the advancement of knowledge.

## Data Availability

The original contributions presented in the study are included in the article/supplementary material, further inquiries can be directed to the corresponding author.
